# Functional investigations on human mesenchymal stem cells exposed to magnetic fields and labeled with clinically approved iron nanoparticles

**DOI:** 10.1186/1471-2121-11-22

**Published:** 2010-04-06

**Authors:** Richard Schäfer, Rüdiger Bantleon, Rainer Kehlbach, Georg Siegel, Jakub Wiskirchen, Hartwig Wolburg, Torsten Kluba, Frank Eibofner, Hinnak Northoff, Claus D Claussen, Heinz-Peter Schlemmer

**Affiliations:** 1 Institute of Clinical and Experimental Transfusion Medicine, University Hospital of Tübingen, Tübingen, Germany; 2 Department of Diagnostic and Interventional Radiology, University Hospital of Tübingen, Tübingen, Germany; 3 Department of Radiology and Nuclear Medicine, Franziskus Hospital, Bielefeld, Germany; 4 Institute of Pathology, University of Tübingen, Tübingen, Germany; 5 Department of Orthopaedics, University Hospital of Tübingen, Tübingen, Germany

## Abstract

**Background:**

For clinical applications of mesenchymal stem cells (MSCs), labeling and tracking is crucial to evaluate cell distribution and homing. Magnetic resonance imaging (MRI) has been successfully established detecting MSCs labeled with superparamagnetic particles of iron oxide (SPIO). Despite initial reports that labeling of MSCs with SPIO is safe without affecting the MSC's biology, recent studies report on influences of SPIO-labeling on metabolism and function of MSCs. Exposition of cells and tissues to high magnetic fields is the functional principle of MRI. In this study we established innovative labeling protocols for human MSCs using clinically established SPIO in combination with magnetic fields and investigated on functional effects (migration assays, quantification of colony forming units, analyses of gene and protein expression and analyses on the proliferation capacity, the viability and the differentiation potential) of magnetic fields on unlabeled and labeled human MSCs. To evaluate the imaging properties, quantification of the total iron load per cell (TIL), electron microscopy, and MRI at 3.0 T were performed.

**Results:**

Human MSCs labeled with SPIO permanently exposed to magnetic fields arranged and grew according to the magnetic flux lines. Exposure of MSCs to magnetic fields after labeling with SPIO significantly enhanced the TIL compared to SPIO labeled MSCs without exposure to magnetic fields resulting in optimized imaging properties (detection limit: 1,000 MSCs). Concerning the TIL and the imaging properties, immediate exposition to magnetic fields after labeling was superior to exposition after 24 h. On functional level, exposition to magnetic fields inhibited the ability of colony formation of labeled MSCs and led to an enhanced expression of lipoprotein lipase and peroxisome proliferator-activated receptor-γ in labeled MSCs under adipogenic differentiation, and to a reduced expression of alkaline phosphatase in unlabeled MSCs under osteogenic differentiation as detected by qRT-PCR. Moreover, microarray analyses revealed that exposition of labeled MSCs to magnetic fields led to an up regulation of CD93 mRNA and cadherin 7 mRNA and to a down regulation of Zinc finger FYVE domain mRNA. Exposition of unlabeled MSCs to magnetic fields led to an up regulation of CD93 mRNA, lipocalin 6 mRNA, sialic acid acetylesterase mRNA, and olfactory receptor mRNA and to a down regulation of ubiquilin 1 mRNA. No influence of the exposition to magnetic fields could be observed on the migration capacity, the viability, the proliferation rate and the chondrogenic differentiation capacity of labeled or unlabeled MSCs.

**Conclusions:**

In our study an innovative labeling protocol for tracking MSCs by MRI using SPIO in combination with magnetic fields was established. Both, SPIO and the static magnetic field were identified as independent factors which affect the functional biology of human MSCs. Further *in vivo *investigations are needed to elucidate the molecular mechanisms of the interaction of magnetic fields with stem cell biology.

## Background

Due to their regenerative and immunomodulatory potential mesenchymal stem cells (MSCs) provide promising opportunities for the therapy of various diseases. For clinical applications of MSCs, labeling and tracking is crucial to evaluate cell distribution and homing. For this purpose positron emission tomography (PET), optical imaging (OI), and magnetic resonance imaging (MRI) were evaluated in various studies [1-5]. Limitations of PET include the poor spatial resolution [6], the radiation of the tracers that results in cell damage, and the short half life time of the tracers used (e.g. F-18, t1/2 110 min). OI also is a very sensitive method widely accepted in small-animal studies [7]. However, due to the limited tissue penetration of the light emitted from the fluorochromes the use of OI is limited to superficial processes. Another problem is the quantification of the signal. High-field MRI (= 1.5 T) in combination with special high-resolution receiver coils and innovative signal strategies might be a compromise for clinical purposes. Advantages of MRI are the lack of radiation exposure, the excellent spatial resolution down to 300 μm and the fact that patient size does not limit the examination. Furthermore, long-term studies over six weeks already have been performed successfully [8]. To label cells for MRI, paramagnetic substances like gadolinium (Gd) DTPA [9-11] or superparamagnetic substances (small/ultrasmall particles of iron oxide, SPIO/USPIO) [12-16] have been used. The latter seem to be more suitable as iron labeling results in larger susceptibility artifacts and iron is less toxic than Gd. Iron-based contrast agents are already FDA and CE approved (Endorem^® ^Guerbet, Paris, France; Resovist^® ^Bayer Schering AG, Berlin, Germany) and under consideration for approval (Sinerem^® ^Guerbet). The major differences of those particles are size (20-120 nm) and coating. SPIO-labeling of MSCs does not affect the viability or differentiation potential of the cells [5,17] but SPIO-labeling can have an impact on the iron metabolism, the migration capacity and the ability of colony formation of MSCs [17-19]. Exposition of cells and tissues to magnetic fields is not only the functional principle of MRI, the magnetic force can also be used for the guided localization of iron-labeled stem cells to desired regions [20,21] or for the seeding of scaffolds with stem cells [22] or for the engineering of 3D tissues by stem cells [23]. Human endothelial progenitor cells and murine macrophages labeled by iron nanoparticles were previously exposed to external magnetic field gradients with different experimental conditions and the formation of the three-dimensional multicellular assemblies have been described [24]. Moreover, magnetic-fluid-loaded liposomes were guided to the near vicinity of human adenocarcinoma prostatic cells by means of a 0.29-T external magnet [25]. Except for sporadic reports on the influence of magnetic fields on hematopoietic progenitor cells [26], neural progenitor cells [27] and myoblasts [28] to date no report exists investigating on possible effects of magnetic fields on iron-labeled and unlabeled human stem cells. In this study an innovative labeling protocol for tracking MSCs by MRI using SPIO in combination with magnetic fields was established. Both, SPIO and the static magnetic field were identified as independent factors which affect the functional biology of human MSCs. Further *in vivo *investigations are needed to elucidate the molecular mechanisms of the interaction of magnetic fields with stem cell biology.

## Results

### Characteriztion of human MSCs

**Figure 1:**
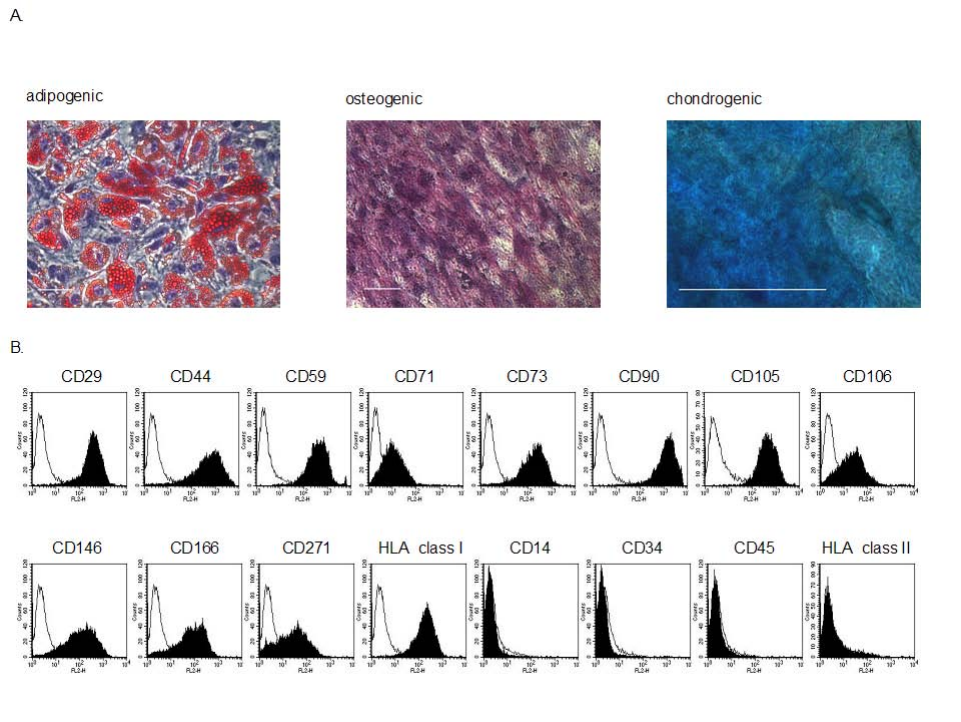
**Characterization of human MSCs**. Specific staining for adipogenesis (lipid vacuoles are stained in red), osteogenesis (alkaline phosphatase is stained in pink-violet) and chondrogenesis (mucopolysaccharides are stained blue-bluish green). Scale bar indicate 50 μm (**A**). FACS analyses of human MSCs: The MSCs are positive for CD29, CD44, CD59, CD71, CD73, CD90, CD105, CD106, CD146, CD166, CD271, and HLA class I and negative for CD14, CD34, CD45, and HLA class II. Black graph: specific antibody, white graph: isotype control (**B**).

After *in vitro *differentiation and specific staining, the human MSCs showed adipogenic, osteogenic and chondrogenic differentiation indicating their plasticity (Figure 1A). Moreover, the human MSCs showed the typical surface epitope pattern: positive for CD29, CD44, CD59, CD71, CD73, CD90, CD105, CD106, CD146, CD166, CD271, and HLA class I and negative for CD14, CD34, CD45, and HLA class II (Figure 1B).

### Cell growth, proliferation and viability

**Figure 2:**
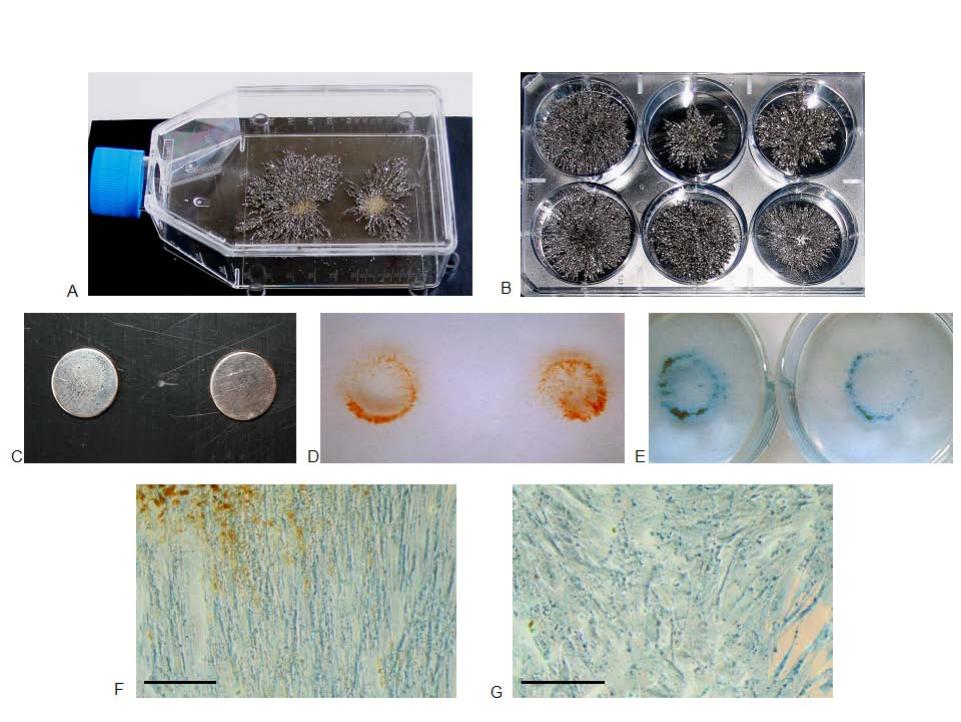
**Magnets, magnetic fields and formation of human MSCs labeled with SPIO**. 3D-visualization of the magnetic fields by cuttings of iron in 75 cm^2 ^culture flasks (**A**) and 6-well plates (**B**). The magnets (**C**) were positioned under the culture flasks and 6-well plates. SPIO-labeled MSCs immediately exposed to the magnetic fields at the time of seeding immediately accumulated in the area over the magnets as identifiable by the red-brown particles (= SPIO) by light microscopy (**D**) and additional blue colour after cell staining with Coomassie blue (**E**). Microphotographs show the orientation of the SPIO-labeled MSCs according to the magnetic flux lines under exposition to magnetic fields (**F**), whereas unlabeled MSCs with or without exposition to magnetic fields and labeled MSCs without exposition to magnetic fields showed the usual clonogenic growth clusters without general order in the culture flasks (**G**). Scale bars indicate 50 μm.

MSCs labeled with SPIO immediately exposed to magnetic fields before adhesion to the plastic surface (JPR-MSC + m(0 h)) initially accumulated in the area directly above the magnets at high density, adhered there guided by the magnetic force and grew out from this area throughout the culture flask or 6-well plates (Figure 2D+E). Unlabeled MSCs immediately exposed to magnetic fields before adhesion to the plastic surface showed no accumulation at the area above the magnets. Labeled or unlabeled MSCs exposed to magnetic fields after adhesion to the plastic surface showed no accumulation at the area above the magnets. MSCs labeled with SPIO and exposed to magnetic fields grew according to the magnetic flux lines (Figure 2F) whereas unlabeled MSCs with or without exposition to magnetic fields and labeled MSCs without exposition to magnetic fields showed the usual clonogenic growth clusters without general order in the culture flasks (Figure 2G).

**Figure 3:**
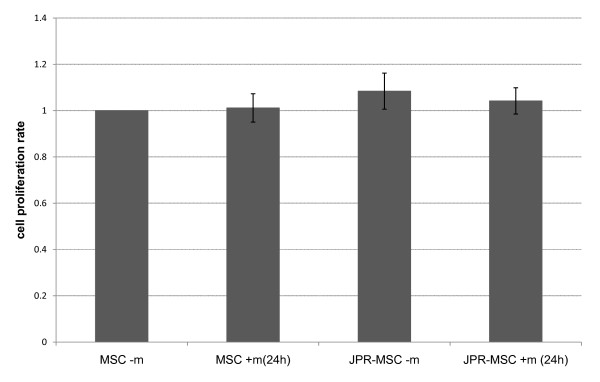
**Proliferation rate of human MSCs**. The proliferation rate of unlabeled MSCs with (MSC + m(24 h)) and without (MSC - m) exposition to magnetic fields and labeled MSCs with (JPR-MSC + m(24 h)) and without (JPR-MSC - m) exposition to magnetic fields showed no significant differences. Error bars: SEM.

The proliferation rate and viability rate of unlabeled MSCs and labeled MSCs with and without exposition to magnetic fields showed no differences (Figure 3 + 4).

**Figure 4:**
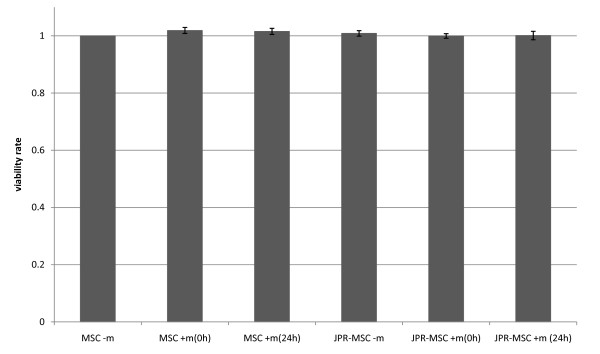
**Viability rate of human MSCs**. The viability rate of unlabeled MSCs with (MSC + m(0 h), MSC + m(24 h)) and without (MSC - m) exposition to magnetic fields and labeled MSCs with (JPR-MSC + m(0 h), JPR-MSC + m(24 h)) and without (JPR-MSC - m) exposition to magnetic fields showed no significant differences. Error bars: SEM.

### Migration capacity

**Figure 5:**
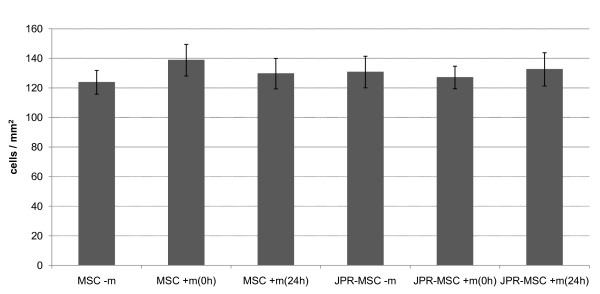
**Migration capacity of human MSCs**. The migration capacity of unlabeled MSCs with (MSC + m(0 h), MSC + m(24 h)) and without (MSC - m) exposition to magnetic fields and labeled MSCs with (JPR-MSC + m(0 h), JPR-MSC + m(24 h)) and without (JPR-MSC - m) exposition to magnetic fields showed no significant differences. Error bars: SEM.

The migration capacity of unlabeled MSCs and labeled MSCs with and without exposition to magnetic fields showed no differences (Figure 5).

### Ability of colony formation

**Figure 6:**
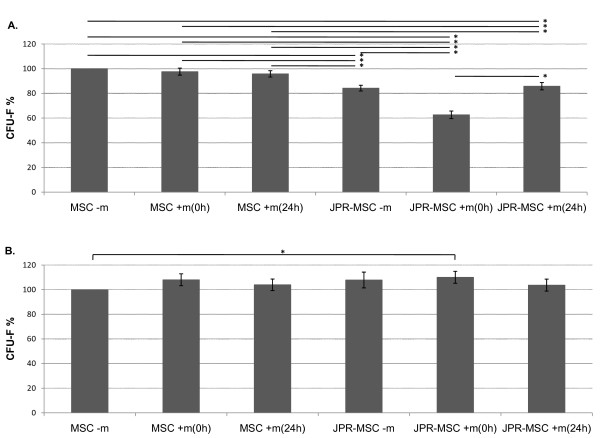
**Colony forming ability of human MSCs**. Colony forming ability of human MSCs **under **exposition to magnetic fields (**A**): The ability of colony formation of labeled MSCs (JPR-MSC - m), (JPR-MSC + m(0 h), (JPR-MSC + m(24 h)) was significantly reduced compared to unlabeled MSCs (MSC - m), (MSC + m(0 h), (MSC + m(24 h)). Exposition to magnetic fields did not influence the ability of colony formation of unlabeled MSCs (MSC - m), (MSC + m(0 h), (MSC + m(24 h)) compared among one another. Immediate exposition of labeled MSCs to magnetic fields (JPR-MSC + m(0 h)) significantly (*) decreased the ability of colony formation compared to labeled MSCs exposed to magnetic fields after 24 h (JPR-MSC + m(24 h)). Colony forming ability of human MSCs **after **exposition to magnetic fields (**B**): 12 days after labeling, the ability of colony formation of labeled MSCs immediately exposed to magnetic fields (JPR-MSC + m(0 h)) was significantly (*) enhanced compared to unlabeled MSCs without exposition to magnetic fields (MSC - m). No other changes of the ability of colony formation of labeled MSCs could be observed in this setting. Error bars: SEM.

A possible influence of the exposition to magnetic fields on the ability of colony formation of labeled and unlabeled MSCs was investigated by two settings:

In the first setting, the colony forming assay was started at the beginning of the exposition to magnetic fields and directly after labeling (= colony forming ability of MSCs under exposition to magnetic fields). Here, the ability of colony formation of labeled MSCs was significantly reduced compared to unlabeled MSCs. The reduced ability of colony formation could be observed on labeled MSCs with and without exposition to magnetic fields. Exposition to magnetic fields did not influence the ability of colony formation of unlabeled MSCs whereas immediate exposition of labeled MSCs to magnetic fields significantly decreased the ability of colony formation compared to labeled MSCs exposed to magnetic fields after 24 h (Figure 6A).

In the second setting, the colony forming assay was started at the end of the exposition to magnetic fields and 12 days after labeling (= colony forming ability of MSCs after exposition to magnetic fields). Here, the ability of colony formation of labeled MSCs immediately exposed to magnetic fields was significantly enhanced compared to unlabeled MSCs without exposition to magnetic fields. No other alteration or reduction of the ability of colony formation of labeled MSCs could be observed in this setting (Figure 6B).

### Gene expression

**Figure 7:**
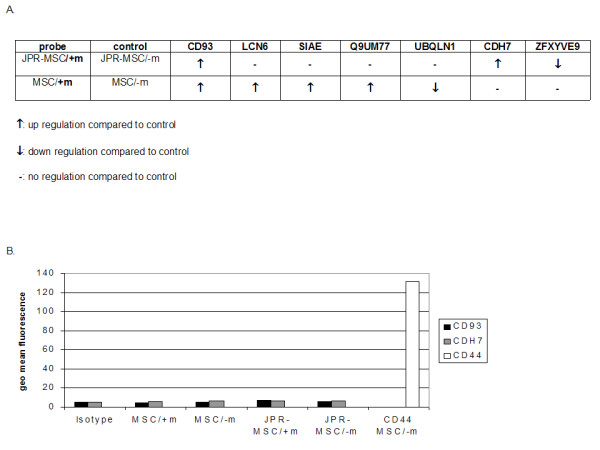
**Influence of exposure to magnetic fields on gene expression of human MSCs**. Expression of mRNA (**A**): Exposition of labeled MSCs to magnetic fields led to an up regulation of CD93 and cadherin 7 and to a down regulation of Zinc finger FYVE domain. Exposition of unlabeled MSCs to magnetic fields led to an up regulation of CD93, lipocalin 6, sialic acid acetylesterase, and olfactory receptor and to a down regulation of ubiquilin 1. Expression of proteins (**B**): No difference of the expression of CD93 and cadherin 7 could be detected by FACS. The very weak expression of CD93 and cadherin 7 is depicted by comparison to the isotype-control and the strongly expressed CD44.

A possible influence of the exposition to magnetic fields on gene expression of labeled and unlabeled MSCs was investigated by microarrays analyzing the mRNA expression of the whole genome. Exposition of labeled MSCs to magnetic fields led to an up regulation of CD93 and cadherin 7 and to a down regulation of Zinc finger FYVE domain. Exposition of unlabeled MSCs to magnetic fields led to an up regulation of CD93, lipocalin 6, sialic acid acetylesterase, and olfactory receptor and to a down regulation of ubiquilin 1 (Figure 7A, Supplemental Table 1). However, on protein level the expression of the extracellulary localized proteins CD93 and cadherin 7 was not affected (Figure 7B).

### Differentiation

**Figure 8:**
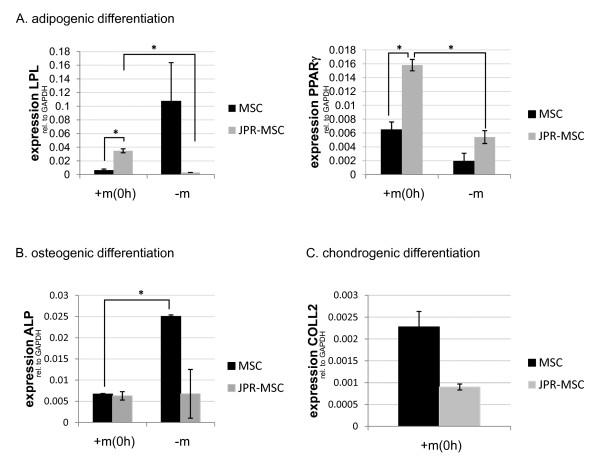
**Influence of exposure to magnetic fields on differentiation of human MSCs**. Exposition to magnetic fields led to a significantly (*) enhanced expression of lipoprotein lipase and peroxisome proliferator-activated receptor gamma under adipogenic differentiation of labeled MSCs (JPR-MSC + m(0 h)) compared to unlabeled MSCs (MSC + m(0 h)) and compared to labeled MSCs without exposition to magnetic fields (JPR-MSC - m). (**A**). Exposition to magnetic fields led to a reduced expression of alkaline phosphatase under osteogenic differentiation of unlabeled MSCs (MSC + m(0 h)) compared to unlabeled MSCs without exposition to magnetic fields (MSC - m) (**B**). Labeled (JPR-MSC + m(0 h)) and unlabeled MSCs (MSC + m(0 h)) showed no significant differences in expression of collagen 2 under exposition to magnetic fields (**C**). Error bars: SEM.

To evaluate the possible influence of the exposition to magnetic fields on the differentiation potential of labeled and unlabeled MSCs the expression of distinct lineage associated markers was quantitatively analyzed by qRT-PCR. Exposition to magnetic fields led to a significantly enhanced expression of lipoprotein lipase and peroxisome proliferator-activated receptor gamma under adipogenic differentiation of labeled MSCs compared to unlabeled MSCs. Moreover, under adipogenic differentiation the expression of lipoprotein lipase and of peroxisome proliferator-activated receptor gamma was significantly enhanced in labeled MSCs exposed to magnetic fields compared to labeled MSCs without exposition to magnetic fields (Figure 8A). This indicates an enhancement of adipogenesis of labeled MSC under the exposition to magnetic fields. Exposition to magnetic fields led to a reduced expression of alkaline phosphatase under osteogenic differentiation of unlabeled MSCs compared to unlabeled MSCs without exposition to magnetic fields (Figure 8B). This indicates an impairment of osteogenesis of unlabeled MSCs under the exposition to magnetic fields. Labeled and unlabeled MSCs showed no significant differences in expression of collagen 2 under exposition to magnetic fields (Figure 8C).

### Electron microscopy and quantification of the TIL

**Figure 9:**
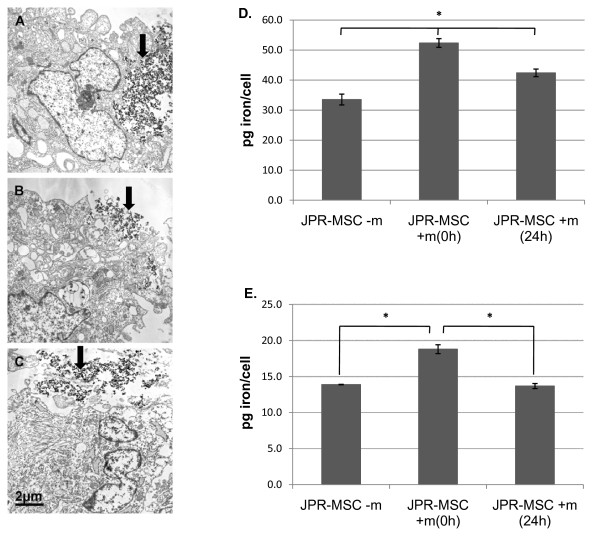
**Electron microscopy and quantification of TIL of human MSCs**. Independently on exposition to magnetic fields, complexes of SPIO (black arrows) were localized on the surface of the MSCs without detectable uptake of SPIO into the cells directly after labeling (**A **= JPR-MSC + m (0 h), **B **= JPR-MSC + m (24 h), **C **= JPR-MSC - m). Seven days after labeling, exposition to magnetic fields led to a significant (*) enhancement of the TIL of MSCs (JPR-MSC + m(0 h), JPR-MSC + m(24 h)) compared to labeled MSCs without exposition to magnetic fields (JPR-MSC - m). Immediate exposition to magnetic fields (JPR-MSC + m(0 h)) was more effective than exposition after 24 h (JPR-MSC + m(24 h)) (**D**). Eleven days after labeling, the highest TIL was again achieved after immediate exposition to magnetic fields (JPR-MSC + m(0 h)), whereas the TIL of the labeled MSCs exposed after 24 h to magnetic fields (JPR-MSC + m(24 h)) was not different from the TIL of the labeled MSCs without exposition to magnetic fields (JPR-MSC - m) (**E**). Error bars: SEM.

Independently on exposition to magnetic fields, complexes of SPIO were localized on the surface of the MSCs without detectable uptake of SPIO into the cells directly after labeling (Figure 9A-C). Seven days after labeling, exposition to magnetic fields led to a significant enhancement of the TIL of MSCs compared to labeled MSCs without exposition to magnetic fields. Immediate exposition to magnetic fields was more effective than exposition after 24 h (Figure 9D). Eleven days after labeling, the highest TIL was again achieved after immediate exposition to magnetic fields, whereas the TIL of the labeled MSCs exposed after 24 h to magnetic fields was not different from the TIL of the labeled MSCs without exposition to magnetic fields (Figure 9E).

### MRI

**Figure 10:**
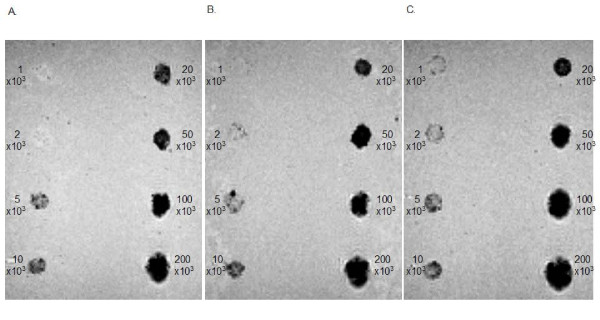
**Magnetic resonance imaging of SPIO-labeled human MSCs**. The lowest detectable cell number was 1 × 10^3 ^labeled MSCs after immediate exposition to magnetic fields (JPR-MSC + m(0 h)) (**C**). The detection limit by MRI of labeled MSCs exposed after 24 h to magnetic fields (JPR-MSC + m(24 h)) was 2 × 10^3 ^MSCs (**B**) and the detection limit by MRI of labeled MSCs without exposition to magnetic fields (JPR-MSC - m) was 5 × 10^3 ^MSCs (**A**).

SPIO labeled MSCs with and without exposition to magnetic fields could be detected by MRI *in vitro *in high quality. The lowest detectable cell number was 1 × 10^3 ^labeled MSCs after immediate exposition to magnetic fields. The detection limit by MRI of labeled MSCs exposed after 24 h to magnetic fields was 2 × 10^3 ^MSCs and the detection limit by MRI of labeled MSCs without exposition to magnetic fields was 5 × 10^3 ^MSCs (Figure 10A-C). According to the TIL, the most effective labeling protocol was using SPIO in combination with immediate exposition to magnetic fields.

## Discussion

MRI has been successfully established detecting *in vivo *MSCs labeled with SPIO [4,5,12]. However, despite initial reports that labeling of MSCs with SPIO is safe without affecting the MSC's biology, recent studies report on influences of SPIO-labeling on metabolism and function of MSCs [17,19]. Moreover, in an animal model of multiple sclerosis the application of SPIO-labeled MSCs led to an aggravation of the symptoms whereas unlabeled MSCs ameliorated the symptoms [29]. Therefore, prior to clinical studies safe and efficient SPIO-based labeling protocols for stem cells have to be established. With respect to the requirements of good manufacturing practice these protocols must include on the one hand comprehensive studies on possible side effects of SPIO-labeling, and on the other hand the optimization of the imaging properties of the SPIO formulation. Exposition of cells and tissues to high magnetic fields is the functional principle of MRI and iron labeled stem cells can be directed by the magnetic force *in vitro *and *in vivo *[21-23].

The imaging properties of labeling protocols using iron nanoparticles correlate to the TIL of the MSCs [19]. In our study we investigated if and how the magnetic force could be used to enhance the amount of magnetic SPIO in/on MSCs in order to optimize the imaging properties. As described previously, the highest TIL of human MSCs without exposition to magnetic fields could be achieved by labeling with SPIO + TA (JPR) and the detection limit by MRI of SPIO labeled rat MSCs + TA (JPR) without exposition to magnetic fields is 5 × 10^3 ^MSCs [19]. In our study we confirmed this detection limit by MRI for human MSCs and immediate exposition of labeled MSCs to magnetic fields maximized the TIL resulting in a minimal detection rate of 1 × 10^3 ^MSCs by a clinical MR scanner. It has to be emphasized that the number of detected cells depends on the sequence used and is not a value in itself. Therefore, comparing protocols with the same sequence as performed in this work does not necessarily reflect an advantage to different labeling protocols using different imaging settings.

The optimized labeling and imaging properties remained stable over a period of eleven days after labeling. Therefore, optimized imaging of the cellular graft would be feasible within the first crucial two weeks after transplantation. The combination of enhanced retention of SPIO in MSCs with more intensive exposition of MSCs to SPIO could be regarded as the major mechanisms of the enhanced efficiency of this labeling protocol. This optimized labeling protocol using magnetic forces in combination with SPIO and TA did not affect cell growth, proliferation, viability and migration capacity of MSCs. The ability of colony formation is a basic property of MSCs reflecting their clonogenic potential related to their stemness [30]. In our study, the ability of colony formation of labeled MSCs under exposition to magnetic fields was significantly reduced compared to unlabeled MSCs, and the reduced ability of colony formation could be observed on labeled MSCs with and without exposition to magnetic fields. These observations correlate to a prior study reporting on the reduced ability of colony formation of MSCs by labeling with JPR [17]. Exposition to magnetic fields did not influence the ability of colony formation of unlabeled MSCs whereas immediate exposition of labeled MSCs to magnetic fields significantly decreased the ability of colony formation compared to labeled MSCs exposed to magnetic fields after 24 h. The fact that the functional influence on the ability of colony formation of MSCs by the magnetic force was only observed on labeled MSCs addresses the issue of the role of SPIO. Whether the observed effect was exclusively caused by the magnetized SPIO or the magnetized SPIO aggravated a possible influence of the magnetic force *per se *remains unanswered but keeping in mind that SPIO are superparamagnetic nanoparticles that are fixed to the MSCs, it is reasonable to assume that the tight growth pattern of SPIO labeled MSCs caused by their accumulation over the area of high density magnetic fields was responsible for the reduced ability of colony formation. The colony forming ability of MSCs 12 days after exposition to magnetic fields was not reduced, the groups showing a reduction of the ability of colony formation under exposition to magnetic fields recovered completely, and the ability of colony formation of labeled MSCs after immediate exposition to magnetic fields was even enhanced. These data confirm the acute influence of labeling and magnetic fields on the ability of colony formation showing that after a time period of 12 days after labeling and 10 days after terminating the exposition to magnetic fields no adversive long term effects on the ability of colony formation may occur.

The exposition to magnetic fields of labeled and unlabeled MSCs influenced the expression of CD93, lipocalin 6, cadherin 7, Zinc finger FYVE domain, sialic acid acetylesterase, olfactory receptor and ubiquilin 1 on mRNA level. However, analyses on protein level showed that the expression of the extracellulary localized proteins CD93, a glycoprotein involved in innate immunity, inflammation and adhesion to endothelium [31-33], and cadherin 7, a protein involved in cell adhesion and in cell dispersion and migration along migratory pathways [34], was not affected. Under the exposition to magnetic fields the adipogenic differentiation of SPIO labeled MSCs was enhanced, whereas no influence of the magnetic fields on the adipogenic differentiation of unlabeled MSCs could be detected. Recent studies showed no influence of SPIO labeling on adipogenic differentiation of MSCs [17,29]. Therefore, the enhanced adipogenic differentiation of the labeled MSCs is most likely caused by the tight growth density of the labeled MSCs over the area of magnetic fields. Under the exposition to magnetic fields the osteogenic differentiation of unlabeled MSCs was reduced indicating an influence of the magnetic fields on the differentiation. In a recent study the membrane potential was identified as an important factor regulating the adipogenic and osteogenic differentiation of MSCs showing that depolarization of MSCs prevents differentiation and hyperpolarization upregulated osteogenic markers [35].

The paramagnetic ions Na^+ ^and K^+ ^are crucial factors in the maintenance of the membrane potential, and their distribution may be influenced by magnetic fields. Kangarlu *et al*. performed systematic analyzes of the effect of an 8.0 T static magnetic field on physiological and/or cognitive function reporting on important changes in the electrocardiogram (ECG) which were related both to the position of the subject in the magnet and to the absolute strength of the magnetic field [36]. Although no cognitive changes and no evidence of detectable changes in body temperature, heart rate, respiratory rate, systolic pressure, and diastolic blood pressure myocardial stiffness index, cardiac output, systolic volume, troponin, and potassium levels were detected [36], the impact on the polarization of a cell mass, as reflected by the changes in the ECG, demonstrates that the static magnetic field has an influence on cell physiology *in vivo*. In our study an innovative labeling protocol with optimized imaging properties for tracking MSCs by MRI using SPIO in combination with magnetic fields was established. The static magnetic field was for the first time identified as an independent factor which can functionally affect the biology and function of human MSCs. Further *in vivo *investigations are needed to elucidate the molecular mechanisms of the interaction of magnetic fields with stem cell biology.

## Conclusions

In our study an innovative labeling protocol for tracking MSCs by MRI using SPIO in combination with magnetic fields was established. Both, SPIO and the static magnetic field were identified as independent factors which affect the functional biology of human MSCs. Further *in vivo *investigations are needed to elucidate the molecular mechanisms of the interaction of magnetic fields with stem cell biology.

## Methods

### Bone marrow preparation

Our study was approved by the local institutional review board (ethical committee). Bone marrow (BM) was taken under sterile conditions from randomly chosen donors (neither metabolic nor neoplastic diseases) with informed consent during orthopaedic operations: From each donor 5 ml of whole BM was collected in a sterile heparinized syringe.

### MSC isolation and cell culture

To isolate MSCs from whole BM we used the density gradient technique as described previously [37]. Briefly, 5 ml bone marrow was resuspended in 10 ml PBS (Cambrex Bio Science, Verviers, Belgium) and laid over 15 ml Lymphoflot (sodium diatrizoate 9.1% [w/v], ficoll 5.7% [w/v]; Biotest AG, Dreieich, Germany). After centrifugation (20 minutes at 1000 × g without braking) the mononuclear cells were harvested, washed twice with PBS, transferred to a 75 cm^2 ^culture flask (Corning Inc., Schiphol-Rijk, Netherlands) and incubated (37°C, 5% humidified CO_2_) with normal medium containing desoxyribonucleotides, ribonucleotides, ultra glutamine 1 (α-MEM, Cambrex Bio Science), 100 I.U./ml Penicillin (Cambrex Bio Science), 100 μg/ml Streptomycin (Cambrex Bio Science) and 10% heat inactivated Fetal Calf Serum (FCS) (Cambrex Bio Science). After 24 hours the non-adherent cells were removed and the adherent cells were cultured and characterized.

### Characterization of human MSCs by *in vitro* differentiation and FACS analysis

MSCs are functionally characterized by in vitro differentiation assays [38]. We evaluated the differentiation potential into three mesenchymal lineages: Adipogenic, osteogenic and chondrogenic differentiation. The MSCs were treated for 21 days with adipogenic medium, osteogenic medium or normal medium (control) as described previously [39,40] (modification: No addition of Amphotericin B): Adipogenic medium contained DMEM + 20% FCS with 1.0 μM dexamethasone (Sigma, Deisenhofen, Germany), 0.5 mM isobutylmethylxanthine (Sigma), 0.2 mM indomethacine (Sigma) and 0.01 mg/ml insulin (Sigma). Osteogenic medium contained normal medium with 10^-8 ^M dexamethasone (Sigma), 0.2 mM ascorbic acid (Sigma) and 10 mM β-glycerolphosphate (Sigma). After 21 days the cell culture was stained with oil red O for in vitro adipogenesis. Briefly, after removal of the medium and washing twice with PBS, 2 ml of 10% formalin were added followed by an incubation time of 30 minutes. After removing the formalin and washing the cell layer with sterile water 2 ml of isopropanol (60%, Bio Whittaker, Verviers, Belgium) were added for 2 minutes. The isopropanol was removed and 2 ml of a filtered working solution of oil red O (3 parts oil red O stock solution [300 mg oil red O powder (Sigma) + 100 ml 99% isopropanol (Sigma)] + 2 parts deionized water) were pipetted onto cells and left there for five minutes. Thereafter the plate was rinsed with tap water and the cells were counterstained with 2 ml haematoxylin (Sigma) for 1 minute. The osteogenic differentiated cells as well as control cells were cytochemically stained for alkaline phosphatase using a commercial staining kit according to the manufacturer's (Cambrex Bio Science) recommendations: after removal of the medium and washing twice with PBS, 2 ml citrate fixative (12.5 ml citrate solution + 32.5 ml acetone + 4 ml 37% formaldehyde) were added for 1 minute, followed by staining with 2 ml alkaline-dye (0.5 ml sodium nitrite + 0.5 ml FRV-alkaline solution + 22.5 ml deionized water + 0.5 ml naphtole AS-BI alkaline solution) for 30 minutes. The cell layer was washed twice and counterstained with 2 ml haematoxylin (Sigma) for 1 minute. Chondrogenic differentiation was performed using a commercially available mesenchymal functional differentiation kit (Cat. Nr. SC006, R&D Systems, Wiesbaden, Germany). The MSC of each population were treated with the chondrogenic differentiation procedure: 250 × 10^3 ^cells were transferred into a 15 ml tube. After centrifugation (200 × g), 1.0 ml basal medium (D-MEM/F-12, Bio Whittaker) and 0.5 ml chondrogenic differentiation medium were added and replaced every 2-3 days. The cell suspension was cultured in the tube forming a solid pellet. After 14 days the chondrocyte pellet was removed, squeezed on a glass slide and stained with 1% Alcian Blue 8GX (Serva, Heidelberg, Germany) in 3% acetic acid (pH 2.5).

FACS analysis was performed with FACScan (BD Biosciences, San Jose, CA, USA) using BD CellQuest Pro software. At subconfluency (1 × 10^6 ^cells) the cells were detached with Accutase™ (PAA Laboratories, Cölbe, Germany)) and washed (PBS + AccuMax™ [PAA Laboratories]). Each probe contained a cell suspension with 5 × 10^5 ^cells in FACS-buffer (PBS + 1% bovine serum albumine [Sigma] + 0.1% FCS [Cambrex Bio Science]). The PE-conjugated antibody (anti-human-CD14, -CD29, -CD34, -CD44, -CD45, -CD59, -CD71, -CD73, -CD90, -CD105, -CD106, -CD146, -CD166, -CD271, HLA class I, HLA class II) was added. After an incubation time of 20 minutes and 2 washing steps the probe was ready for analysis. All antibodies, except for anti-human-CD271 (Miltenyi Biotech, Bergisch Gladbach, Germany), were from BD Biosciences.

### Contrast Media and cell labeling

Resovist^® ^(SHU 555A, Bayer Schering AG, Berlin, Germany), an MRI contrast agent already approved for clinical use in Europe, Japan and Australia, is organ-specific and used for liver imaging [41-43]. It consists of superparamagnetic iron oxide (SPIO) nanoparticles (4-6 nm) [44] coated with carboxydextran (mean hydrodynamic diameter 60 nm), which is accumulated by phagocytosis in *von Kupffer *cells. For an enhanced iron loading of the cells, the transfection reagent (TA) jetPEI™, a linear polyethylenimine (PolyPlus Transfection, Illkirch, France) was used. Cell labeling was performed for 4 h with 60 μg/ml Resovist^® ^in combination with jetPEI™ in 6-well-plates seven days after seeding of 5 × 10^4 ^cells as described previously [19]. First, 10 μl of the TA in a total volume of 50 μl PBS was carefully added to 50 μl of the Resovist^® ^solution, mixed and preincubated for 30 min. The following incubation was carried out in the incubator at standard conditions (37°C, 5% humidified CO_2_). After the labeling, cells were washed three times with PBS, harvested and processed for further plating with or without magnets.

### Magnets

Magnets (magnetic material: NeoSint N33H (alloy of neodymium, dysprosium, boron, cobalt and iron), remanence: > 1.140 mT, magnetic field force on the surface: 600 mT, coercive force, BHC = > 851 kA/m, JHC = > 1.353 kA/m, max. residual energy > 247 kJ/m3, magnetisation direction: axial, coating: nickel, diameter: 15 mm, thickness: 3 mm (ppm materials GmbH, 70794 Filderstadt, Germany)) were fixed on plastic dishes and the 75 cm^2 ^culture flasks and 6-well plates were firmly positioned with direct contact above the magnets (Figure 2A-C). The 75 cm^2 ^culture flasks were used for the proliferation and viability assays, the transmission electron microscopy, the quantification of the total iron load and the adipogenic and osteogenic differentiation analyses, the 6-well plates were used for the colony formation analyses and the microarray experiments. The following conditions were investigated: unlabeled MSCs without exposition to magnetic fields (MSC - m), unlabeled MSCs with exposition to magnetic fields 24 hours after seeding into the 75 cm^2 ^culture flasks and 6-well plates (MSC + m(24 h)), unlabeled MSCs with exposition to magnetic fields immediately after seeding into the 75 cm^2 ^culture flasks and 6-well plates and before adhesion to the plastic surface (MSC + m(0 h)), SPIO-labeled MSCs without exposition to magnetic fields (JPR-MSC - m), SPIO-labeled MSCs with exposition to magnetic fields 24 hours after seeding into the 75 cm^2 ^culture flasks and 6-well plates (JPR-MSC + m(24 h)), and SPIO-labeled MSCs with exposition to magnetic fields immediately after seeding into the 75 cm^2 ^culture flasks and 6-well plates and before adhesion to the plastic surface (JPR-MSC + m(0 h)). The exposition to the magnetic fields continued until the end of the respective experiments.

### Proliferation assays and determination of viability

Proliferation assays were performed in 75 cm^2 ^culture flasks positioned with direct contact above the magnets. Labeled and control cells were seeded at 3 × 10^5 ^cells/flask in standard medium (α-MEM, 100 I.U./ml Penicillin, 100 μg/ml Streptomycin and 10% Fetal Calf Serum). The incubation was carried out in the incubator at standard conditions (37°C, 5% humidified CO_2_). The labeled and unlabeled MSCs were exposed to magnetic field immediately after seeding, 24 hours after seeding into the 75 cm^2 ^culture flasks or there was no exposure (control group).

Medium was changed every 3 days. At subconfluence (90%) the cells were detached with Accutase (PAA Laboratories, Cölbe, Germany) and counted with a CASY^®^2 Analyser (CASY^®^2-Cell Counter and Analyser System, Model TT, Roche Diagnostics, Mannheim, Germany). The experiments were performed in triplicates.

Cellular viability after the different incubation conditions was examined with the help of a CASY^®^2 Analyser according to the ECE method described by Lindl et al [45] and the viability-SOP of the manufacturer.

### Quantification of the total iron load (TIL)

After reaching subconfluence MSC + m(24 h), MSC + m(0 h) and (MSC - m), all Resovist^®^/jetPEI™ labeled, were washed three times with PBS, harvested with Accutase and counted with a CASY^®^2 Analyser. A cell pellet containing 1 × 10^5 ^cells was dried for 2 hours at 80°C. Then samples were incubated overnight at room temperature and another 2 hours at 60°C in perchloric and nitric acid at a 3:1 ratio to completely digest the cells and expose iron oxide from the dextran coated nanoparticles. For photometric determination of the TIL, a Ferrozine-based spectrophotometric assay (Eisen Ferene S Plus^®^, Rolf Greiner Biochemica, Flacht, Germany) was used. Fe^2+ ^forms a blue complex with Ferene which can be measured at 595 nm. The extinction of the sample relates directly to the iron concentration, calculated with the help of a defined standard. All experiments were performed in triplicates.

### Transmission electron microscopy

To assess the uptake and localization of the SPIO nanoparticles and their possible influence on the cellular ultrastructure, electron microscopy was performed. Cells grown as described above in the proliferation section and treated as indicated were fixed in 2.5% glutaraldehyde (Paesel-Lorei, Frankfurt, Germany) buffered with 0.1 M cacodylate buffer (pH 7.4), postfixed in 1% OsO_4 _in 0.1 M cacodylate buffer and scraped off the plastic. The pellet was then dehydrated in an ethanol series (50%, 70%, 96%, 100%). The 70% ethanol was saturated with uranyl acetate for contrast enhancement. Dehydration was completed in propylene oxide. The specimens were embedded in Araldite (Serva). Ultrathin sections were produced on a FCR Reichert Ultracut ultramicrotome (Leica, Bensheim, Germany), mounted on pioloform-coated copper grids, contrasted with lead citrate and analyzed and documented with an EM 10A electron microscope (Zeiss, Oberkochen, Germany).

### *In vitro* MRI

An agar matrix was used as suitable environment for imaging SPIO labeled MSCs. The agar solution (1%) was boiled and embedded in nonferromagnetic boxes before becoming stable. By using a special stamp a series of identical cone shaped cavities was created in the agar block. For MR measurement cell numbers from one thousand to two hundred thousand Resovist^® ^labeled MSCs were used (JPR-m; JPR + m(24 h); JPR + m(0 h)). Cells were centrifuged at 200 × g for 5 minutes, dissolved in 8% gelatine (20 μl) and implanted into the cone shaped cavities within the agar matrix. After solidification of the gelatine the hollows were closed with agar. Thus, it was possible to achieve a homogenous distribution of the target cells in a defined volume of 20 μl within a homogenous agar block. Cells were scanned in a clinically used MR scanner at 3.0 T (Magnetom Trio, Siemens Healthcare, Erlangen, Germany) using the wrist coil of the manufacturer. Imaging was performed with a conventional 3D gradient echo sequence with a Field-of-View (FoV) of 83 × 120 and a matrix size of 176 × 256, resulting in a resolution of 0.21 mm. The echo time (TE) was chosen as the variable parameter and was varied between 5 ms and 15 ms in steps of 5 ms with a repetition time (TR) of 70 ms and a flip angle of 14° according to the T_1_-value of the agarose gel of 2000 ms. Further parameters were: readout bandwidth (BW) 220 Hz/Px, 32 slices with a slice thickness of 0.5 mm. The acquisition time was then for one average about 6.5 minutes. Signal extinction was described by measuring the dimensions of the signal artifacts in all directions.

### Migration assays

The migratory ability of labeled and control MSCs was investigated with cells which finished the proliferation assay. Every six conditions were analyzed (JPR-MSC - m; JPR-MSC + m(24 h); JPR-MSC + m(0 h) and MSC - m; MSC + m(24 h); MSC + m(0 h). There was no magnetic exposure during migration assay.

The MSCs were tested in 24-well compound chambers (Falcon, Becton Dickinson, Franklin Lakes, NJ, USA) with 8 μm pore membrane inserts. Cells were washed three times with PBS, harvested and finally seeded at a density of 20.000 cells in the membrane inserts with 0.5 ml cell culture medium containing 20% FCS. 0.8 ml medium containing 20% FCS and 25 ng/ml PDGF-BB (R&D Systems), serving as a chemoattractant, was added to the lower compartment of the plate. After incubating the plates for 24 h at 37°C in a humidified 5% CO_2 _atmosphere, the membrane inserts were fixed (ethanol 70%, formaldehyde 3.5%, 10 min. respective) and stained with Coomassie blue after mechanically removing the cells attached to the upper surface. The cells on the lower side of the inserts were counted with the help of a 100× magnification light microscope (Zeiss, Göttingen, Germany) using a Fuchs-Rosenthal counting-chamber. For each condition 5 membranes have been investigated.

### Colony forming assays

The clonogenic activity of the cells was determined with the help of colony forming assays. There were two different setups, under exposition to magnetic field and after exposition to magnetic field. Every six conditions were analyzed (JPR-MSC - m; JPR-MSC + m(24 h); JPR-MSC + m(0 h) and MSC - m; MSC + m(24 h); MSC + m(0 h).

The cells were seeded at 250 cells/well in 2 ml medium in 6-well plates (Falcon), resulting in 12 wells per condition. The plates were incubated at 37°C in a 5% CO_2 _atmosphere without changing medium. After 10 days, the assays were stopped and the cells were fixed with 3.5% formaldehyde and 70% ethanol and subsequently stained with Coomassie blue. The total number of colonies exceeding 50 cells per colony was counted by light microscopy (Zeiss). Because of the intense inter-donator variation of the clonogenic activity of the MSCs, data are given as percentage with respect to the controls.

### Investigations on gene and protein expression

Gene expression analyses were performed with Agilent Whole Human Genome Microarrays, 4 × 44 K, Two Color (Miltenyi Biotec).

The MSCs were seeded at 1 × 10^5 ^cells/well in 6-well plates (Falcon). The plates were incubated at 37°C in a 5% CO_2 _atmosphere, as far as they reached subconfluency (90%), and labeled with standard protocol above, following exposure to magnetic field for 24 h (JPR-MSC - m; JPR-MSC + m(0 h) and MSC - m; MSC + m(0 h)). Cells were washed three times with PBS, harvested with Accutase, frosted in liquid nitrogen and stored at -80°C until RNA isolation

The microarrays were performed following the manufacturer's protocol. RNA was isolated using standard RNA extraction protocols (NucleoSpin^® ^RNA II, Macherey-Nagel, Düren, Germany). The RNA samples were quality-checked via the Agilent 2100 Bioanalyzer platform (Agilent Technologies, Waldbronn, Germany). The quality of the isolated RNA was checked in a gel image and an electropherogram using the Agilent 2100 Bioanalyzer expert software. In addition to the visual control, the software allows the generation of a RNA Integrity Number (RIN) to check integrity and overall quality of total RNA samples. All samples showed sufficient quality for gene expression profiling experiments.

For the linear T7-based amplification step, 1 μg of each total RNA sample was used. To produce Cy3- and Cy5-labeled cRNA, the RNA samples were amplified and labeled using the Agilent Low RNA Input Linear Amp Kit (Agilent Technologies) following the manufacturer's protocol. Yields of cRNA and the dye-incorporation rate were measured with the ND-1000 Spectrophotometer (NanoDrop Technologies, Wilmington, USA).

The hybridization procedure was performed according to the Agilent 60-mer oligo microarray processing protocol using the Agilent Gene Expression Hybridization Kit (Agilent Technologies). Briefly, 825 ng of the corresponding Cy3- and Cy5-labeled fragmented cRNA were combined and hybridized overnight (17 hours, 65°C) to Agilent Whole Human Genome Oligo Microarrays 4 × 44 K using Agilent's recommended hybridization chamber and oven. Finally, the microarrays were washed once with 6× SSPE buffer containing 0.005% N-lauroylsarcosine for 1 min at room temperature followed by a second wash with pre-heated 0.06× SSPE buffer (37°C) containing 0.005% N-lauroylsarcosine for 1 min. The last washing step was performed with acetonitrile for 30 sec.

Fluorescence signals of the hybridized Agilent Oligo Microarrays were detected using Agilent's DNA microarray scanner (Agilent Technologies).

The Agilent Feature Extraction Software (FES) was used to read out and process the microarray image files. The software determines feature intensities and ratios (including background subtraction and normalization), rejects outliers and calculates statistical confidences (p-values). For determination of differential gene expression FES derived output data files were further analyzed using the Rosetta Resolverâ gene expression data analysis system (Rosetta Biosoftware, Seattle, USA). This software offers the possibility to visualize the results of the data analysis in a double-log scatter plot.

FACS analysis of CD93 and Cadherin7 (CDH7) epitope pattern was performed for the conditions JPR-MSC - m; JPR-MSC + m(0 h) and MSC - m; MSC + m(0 h). 10 × 10^5 ^cells were incubated for 30 min with unconjugated anti-CD93 antibody (R × D Systems) or anti-CDH7 antibody (Sigma), followed by a respective Alexa488 secondary antibody staining. A FACScan (BD Biosciences) and BD CellQuest Pro software were used.

To evaluate the differences in differentiation potential of the MSCs, adipogenic, osteogenic and chondrogenic differentiation of unlabeled and labeled MSCs with and without exposition to magnetic fields was performed.

Differentiation assays under magnetic exposition were carried out in 75 cm^2 ^culture flasks for adipogenic and osteogenic differentiation, and in 15 ml Falcon tubes for chondrogenic differentiation, all directly above the magnets.

Labeled and control cells were seeded at 3 × 10^5 ^cells in standard medium for two days. The incubation was carried out in the incubator under standard conditions. The labeled and unlabeled MSCs were exposed to magnetic field immediately after seeding, or there was no exposure (control group). After two days the medium was replaced with differentiation media, as described above. Medium was changed every 3 days. After 21 days the cells were detached with Accutase, counted with a CASY^®^2 Analyser, and stored in RLT-buffer (Qiagen, Hilden, Germany) at -80°C.

To quantify the tri-lineage differentiation potential of the MSCs, quantitative Reverse Transcription-Polymerase Chain Reaction Analysis (RT-PCR) was performed as described previously [17]: Briefly, total ribonucleic acid (RNA) was extracted from adipogenic, osteogenic and chondrogenic differentiated MSCs using RNeasy mini spin columns (Qiagen). Reverse transcription was performed by Transcriptor First Strand cDNA Synthesis Kit (Roche Diagnostics), using anchored-oligo(dT)_18 _primer. The expression of lineage-specific genes was determined by ready-to-use amplification primer mixes for RT-PCR (search-LC, Heidelberg, Germany) and the LightCycler™ Instrument (Roche Diagnostics). In addition, the expression of Glyceraldehyde 3-phosphate dehydrogenase (GAPDH) was determined in the same way in all samples. For relative quantification of the gene expression, the expression of each target gene was normalized to the expression of GAPDH in the same sample.

### Statistics/Data analysis

Following assessment of normal distribution, statistical significance was tested by Student's t test. The data are presented in mean ± standard error of mean (SEM), p < 0.05 was considered significant (*).

## Abbreviations

MSCs: mesenchymal stem cells; MRI: magnetic resonance imaging; SPIO: small particles of iron oxide; TIL: total iron load; PET: positron emission tomography; OI: optical imaging; Gd: gadolinium; TA: transfection agent; JPR: JetPei™ + Resovist^®^; BM: Bone marrow; T: Tesla; CDH7: Cadherin7; DTPA: diethylentriamine penta acetic acid; GAPDH: Glyceraldehyde 3-phosphate dehydrogenase; PPAR-γ: peroxisome proliferator-activated receptor-γ; ECG: electrocardiogram

## Competing interests

The authors declare that they have no competing interests.

## Authors' contributions

RS designed and co-ordinated the study, performed the statistical analyses, participated in the isolation and characterization of the MSCs, and wrote the manuscript. RB participated in the design of the study and in the co-ordination of the study, cultured the MSCs under the different conditions, and performed the colony forming assays and migration assays. RK participated in the writing of the manuscript, performed the cell labeling, the determination of the TIL, and the FACS analyses, and participated in the co-ordination of the study. GS performed the quantification of the mRNA expression. JW participated in the design of the study. HW performed the TEM. TK provided the human BM for the isolation of the MSCs. FE performed the in vitro MRI. HN participated in the writing of the manuscript and in the co-ordination of the study. CDC participated in the writing of the manuscript and in the co-ordination of the study. HPS participated in the design of the study, in the interpretation of the data, and in the writing of the manuscript. All authors read and approved the final manuscript.

## Supplementary Material

Additional file 1 **Supplemental Table 1. Regulation of mRNA expression by magnetic fields**. The table gives detailed background information as sequence ID, sequence name, sequence description, Log(Ratio), fold change, and p-value on the microarray data shown in Figure 7. Click here for file
