# Supplementary material for: Functional investigations on human mesenchymal stem cells exposed to magnetic fields and labeled with clinically approved iron nanoparticles
Source: BMC Cell Biol. 2010 Apr 6;11:22. doi: 10.1186/1471-2121-11-22 (PMC2871263; doi:10.1186/1471-2121-11-22)
Supplement: Additional file 1 — Supplemental Table 1. Regulation of mRNA expression by magnetic fields. The table gives detailed background information as sequence ID, sequence name, sequence description, Log(Ratio), fold change, and p-value on the microarray data shown in Figure 7. [file 1471-2121-11-22-S1.DOC]

**Supplemental Table 1**

**Regulation of mRNA expression by magnetic fields**

JPR-MSC/**+m** vs. JPR-MSC/-m

| Sequence ID | Sequence Name | Sequence Description | Log(Ratio) | Fold Change | P-value |
| --- | --- | --- | --- | --- | --- |
| 651345 | CD93 | Homo sapiens CD93 molecule (CD93),  mRNA [NM_012072] | 0.67937 | 4.77937 | 0.00728 |
| 651352 | CDH7 | Homo sapiens cadherin 7, type 2 (CDH7), transcript variant b, mRNA [NM_004361] | 0.36909 | 2.33933 | 0.00151 |
| 677875 | ZFYVE9 | Homo sapiens zinc finger, FYVE domain containing 9 (ZFYVE9), transcript variant 2, mRNA [NM_007323] | -0.42645 | -2.6696 | 0.00113 |

MSC/**+m** vs. MSC/-m

| Sequence ID | Sequence Name | Sequence Description | Log(Ratio) | Fold Change | P-value |
| --- | --- | --- | --- | --- | --- |
| 651345 | CD93 | Homo sapiens CD93 molecule (CD93),  mRNA [NM_012072] | 0.73045 | 5.37589 | 0.00067 |
| 646756 | LCN6 | Homo sapiens lipocalin 6 (LCN6),  mRNA [NM_198946] | 0.67188 | 4.69766 | 0.00179 |
| 651278 | SIAE | Homo sapiens sialic acid acetylesterase (SIAE),  mRNA [NM_170601] | 0.32073 | 2.09281 | 0.00369 |
| 660722 | THC2423326 | Q9UM77 (Q9UM77) Olfactory receptor 17-210, complete [THC2423326] | 0.4279 | 2.67854 | 0.00603 |
| 678977 | UBQLN1 | Homo sapiens ubiquilin 1 (UBQLN1), transcript variant 1, mRNA [NM_013438] | -0.37354 | -2.36344 | 1.85E-07 |
